# Selective signatures and high genome-wide diversity in traditional Brazilian manioc (*Manihot esculenta* Crantz) varieties

**DOI:** 10.1038/s41598-022-05160-8

**Published:** 2022-01-24

**Authors:** Alessandro Alves-Pereira, Maria Imaculada Zucchi, Charles R. Clement, João Paulo Gomes Viana, José Baldin Pinheiro, Elizabeth Ann Veasey, Anete Pereira de Souza

**Affiliations:** 1 Departamento de Biologia Vegetal, Instituto de Biologia, Universidade Estadual de Campinas (UNICAMP), Av. Cândido Rondon, 400, Cidade Universitária, CP: 6010, Campinas, SP 13083‐875 Brazil; 2 Centro de Biologia Molecular e Engenharia Genética, Universidade Estadual de Campinas (UNICAMP), Av. Cândido Rondon, 400, Cidade Universitária, CP: 6010, Campinas, SP 13083‐875 Brazil; 3 Agência Paulista de Tecnologia Dos Agronegócios (APTA), Pólo Centro-Sul. Rodovia SP 127, km 30, Piracicaba, SP 13400-970 Brazil; 4 Instituto Nacional de Pesquisas da Amazônia (INPA), Av. André Araújo, 2936, Petrópolis, Manaus, AM 69067-375 Brazil; 5 Department of Crop Sciences, University of Illinois at Urbana-Champaign (UIUC), AW-101 Turner Hall, 1102 South Goodwin Avenue, Urbana, IL 61801-4798 USA; 6 Departamento de Genética, Escola Superior de Agricultura “Luiz de Queiróz”, Universidade de São Paulo (ESALQ/USP), Av. Pádua Dias, 11, Piracicaba, SP 13400-970 Brazil

**Keywords:** Genetic variation, Plant domestication, Agricultural genetics, Molecular ecology

## Abstract

Knowledge about genetic diversity is essential to promote effective use and conservation of crops, because it enables farmers to adapt their crops to specific needs and is the raw material for breeding. Manioc (*Manihot esculenta* ssp. *esculenta*) is one of the world’s major food crops and has the potential to help achieve food security in the context of on-going climate changes. We evaluated single nucleotide polymorphisms in traditional Brazilian manioc varieties conserved in the gene bank of the Luiz de Queiroz College of Agriculture, University of São Paulo. We assessed genome-wide diversity and identified selective signatures contrasting varieties from different biomes with samples of manioc’s wild ancestor *M*. *esculenta* ssp. *flabellifolia*. We identified signatures of selection putatively associated with resistance genes, plant development and response to abiotic stresses that might have been important for the crop’s domestication and diversification resulting from cultivation in different environments. Additionally, high neutral genetic diversity within groups of varieties from different biomes and low genetic divergence among biomes reflect the complexity of manioc’s evolutionary dynamics under traditional cultivation. Our results exemplify how smallholder practices contribute to conserve manioc’s genetic resources, maintaining variation of potential adaptive significance and high levels of neutral genetic diversity.

## Introduction

Food security—the regular access to enough high-quality food with sufficient protein and energy—is one of the major goals of the United Nations’ 2030 Agenda for Sustainable Development to promote a fairer world^1^. It is, however, an enormous challenge due to the accelerated increase in the world’s human population and on-going global climate changes^2,3^. Better strategies to conserve and use crop diversity are effective ways to address this issue, and, although often regarded as descriptive, the study of genetic diversity is fundamental to this end^4,5^.

Genetic diversity is used by farmers to adapt their crops to current and future climate changes and is also the raw material of formal breeding^5,6^. The maintenance of agrobiodiversity provides humans with a wealth of intraspecific crop diversity selected in different cultural and geographical contexts^7,8^. This agrobiodiversity is associated with valuable traditional knowledge and practice systems, which play a key role to conserve biological and cultural diversity^9^. Additionally, the management of landraces with high genetic diversity by farming communities has collaborated to keep reasonable levels of food production in many areas of the developing world^8^. However, factors such as globalization, degradation of natural landscapes, changes in agricultural production systems, and landrace displacement by modern cultivars threaten the conservation of plant genetic resources^5,8^.

Manioc (*Manihot esculenta* Crantz) is currently one of the major food crops, ranking eighth in estimated global production^10^, and its roots are the main source of energy for more than 800 million people^11^. Manioc is also widely known as cassava, but here we use the former term because it derives from a Tupi word that means cultivated plant, while the latter derives from an Arawak word that means bread^11,12^. The crop has an immense diversity of varieties which are cultivated around the Tropics, mainly by low-income smallholder farmers^13,14^, and it is considered one of the most promising crops to promote food security in developing countries^14^. This is because manioc is well-adapted to marginal areas with poor soils and can be produced efficiently even on small scales, with low inputs and without mechanization^14^.

Morphological and genetic evidence support the hypothesis that manioc was domesticated from *M. esculenta* ssp. *flabellifollia* in southwestern Amazonia^15–20^, although there is still some controversy^11^. Domestication may have started as early as 10,000 years before present (ybp)^16^, in what is now the Brazilian state of Rondônia and adjacent regions. Populations of ssp. *flabellifolia* currently occur in the Amazonia-Cerrado ecotones of southern Amazonia, as well as on the Guiana shield^15^. The wild progenitor grows as highly branched shrubs in open vegetation or as climbing vines amid denser vegetation, while cultivated manioc grows as little-branched shrubs^21^. After its initial domestication, manioc was probably available in most parts of the Neotropics by 6500 ybp^22,23^. Manioc started to be globally spread after the European conquest of South America in the sixteenth century, which introduced the crop in Africa, tropical Asia and Oceania^13^.

Cultivated manioc has two major groups of landraces that differ in their contents of cyanogenic compounds (in the whole plant, but especially in the edible roots). Sweet manioc has lower cyanogenic potential (< 100 ppm fresh weight) than bitter manioc (> 100 ppm fresh weight), but the variation in the content of cyanogenic compounds is continuous across these two groups^24^. Sweet manioc can be safely consumed after simple processing (e.g., peeling and cooking), while bitter manioc needs more elaborate detoxification (e.g., peeling, soaking, grating, and roasting)^25^. Although sweet and bitter manioc cannot be separated by morphological traits, they are genetically divergent and farmers’ traditional knowledge categorize them clearly^13^.

Manioc is a clonal crop, but its evolutionary dynamics is much more complex than the establishment of varieties consisting of unique clones. Under traditional cultivation, farmers propagate manioc varieties by stem-cuttings, but the plants can produce flowers^11^. Manioc is allogamous, and crossings may occur between plants from different varieties producing fruits that disperse sexual seeds in the swiddens^13^. The seeds have elaiosomes that attract ants, which further disperse and bury them^26^. The sexual seeds in the soil seed banks may sprout when the swidden is cleared, or the vegetation of an abandoned field is burnt to start a new cycle of cultivation^27^. Farmers may consciously or inadvertently let sexual seedlings grow in the swiddens until harvesting time, when they may decide to use stem-cuttings of sexual plants for clonal propagation^28,29^. Farmers may either incorporate these stem-cuttings into an existing variety or start a new variety, increasing the crop’s genetic diversity^30^. This management of high genetic diversity extends beyond family units into extensive exchange networks of cultivated varieties^31,32^. These networks vary according to the cultural context, but are a common feature of traditional cultivation in Amazonia and throughout the world^33–36^. By exchanging varieties that were selected and managed in the same or different regions, traditional farmers maintain high levels of genetic diversity in the crop at both local and wider geographical scales^36,37^.

The evolutionary dynamics outlined above is typical of manioc cultivation in Amazonia, but the crop’s dispersals around the world were not always accompanied by cultural appropriation, i.e., not all the original aspects of its cultivation can be observed outside Amazonia^13^. In the Neotropics, bitter landraces predominate where manioc is the major staple crop cultivated in swiddens far from household units, while sweet landraces predominate where manioc is part of multi-crop systems and is often cultivated in backyards^13^. These same patterns may be observed in most parts of Africa, but are much more variable in Asia and Oceania^37,38^. Farmer’s interest in experimenting with sexual seedlings also seems to vary greatly across locations outside the Neotropics^13^. Variable degrees of cultural appropriation may also influence the farmers’ ability to properly detoxify bitter manioc before safe consumption, which contributes to epidemics of *Konzo*, a chronic paralytic disease, in some African regions^13^. These differences in the crop’s management influence how manioc evolves under distinct geographical and cultural contexts and have a significant impact in the crop’s potential to contribute to food security^39^.

Genetic studies have greatly contributed to our understanding about the evolution of the crop^29,34,40^ and to support breeding^41–43^. More recently, approaches assessing genome-wide diversity of domesticated varieties and wild relatives^44–46^ advanced rapidly after the release of a genome for manioc^47^. Indeed, the characterization of genome-wide diversity collaborated tremendously for the valorization and utilization of wild species and cultivated varieties conserved in gene banks throughout the world^48–50^. Given the agronomic importance of manioc, these studies were mostly developed to support breeding. However, the genomic approaches also offer opportunities to further investigate evolutionary aspects related to the crop’s domestication and diversification^51,52^.

**Figure 1:**
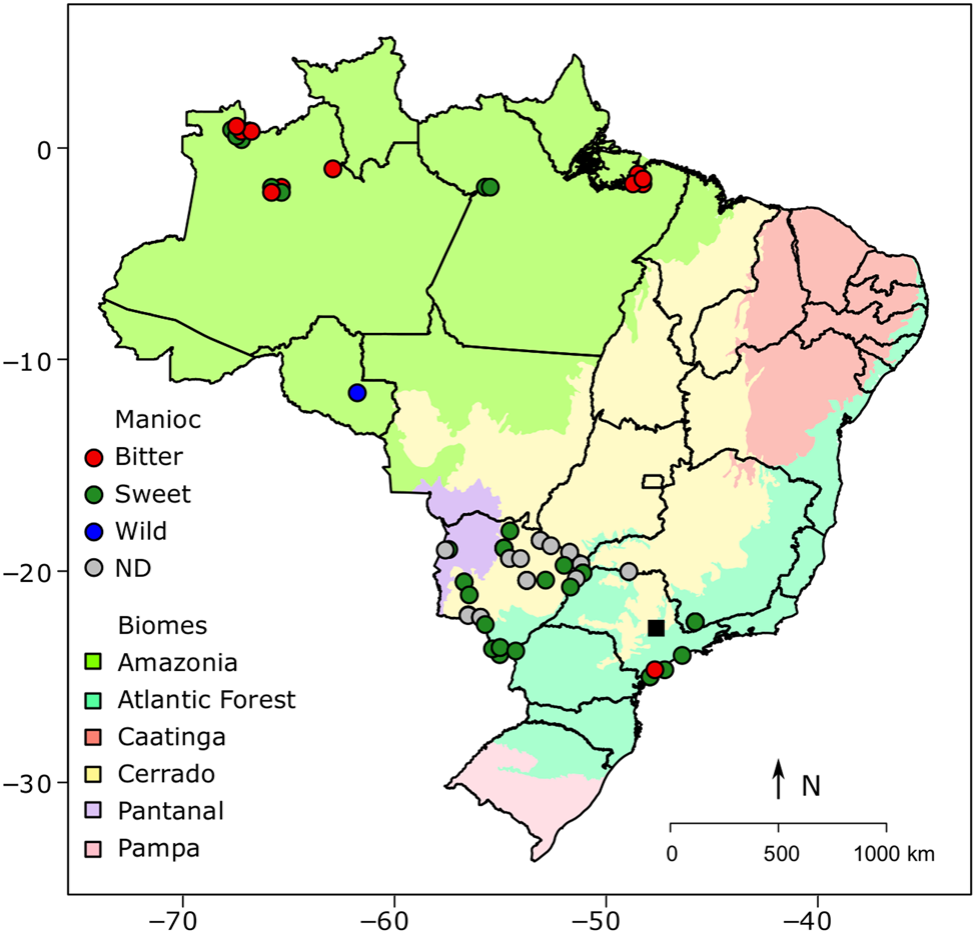
Map of Brazil showing the geographical locations of the municipalities in which manioc (*Manihot esculenta*) samples were originally collected (some points were slightly moved for easier visualization). The black square indicates the location of the gene bank at the Luiz de Queiroz College of Agriculture, Piracicaba, São Paulo, Brazil. ND = no data on toxicity. The map was drawn with maptools 0.8–36 (https://CRAN.R-project.org/package=maptools).

In this context, we assessed selective signatures and the genome-wide diversity of manioc varieties from different Brazilian biomes (Fig. 1, Supplementary Table S1), based on single nucleotide polymorphisms (SNP). The varieties are conserved at the Luiz de Queiroz College of Agriculture gene bank, Brazil, and are compared with wild samples collected in the center of manioc domestication. We performed genome scans to identify SNPs with putative signatures of selection and discuss their potential relevance to the domestication and diversification processes associated with cultivation in different environmental contexts. We aimed to generate novel information about the evolution of manioc in its country of origin and to contribute to better management of the genetic resources of this globally important crop.

## Results

### SNP genotyping

Sequencing of the two ddGBS libraries resulted in 198,017,706 (*NsiI*-*MspI*) and 240,176,492 (*PstI*-*MseI*) raw reads. After demultiplexing and quality filtering, 153,858,282 reads for *NsiI*-*MspI* (mean of 1,672,372.6 reads per sample ± 406,184.6 SD) and 118,476,082 reads for *PstI*-*MseI* (mean of 1,287,783.5 reads per sample ± 540,792.9 SD) were used for SNP identification. The *NsiI*-*MspI* library resulted in 4790 SNPs (34.8 mean sequence depth per locus ± 21.3 SD, 2.2% of missing data) while the *PstI*-*MseI* library resulted in 10,121 SNPs (29.5 mean sequence depth per locus ± 16.8 SD, 4.8% of missing data). After merging these data sets and pruning markers with high LD we obtained a final set of 11,782 high-quality SNPs (31.2 mean sequence depth per locus ± 18.5 SD, 4% of missing data) (Supplementary Figure S1, Table S2).

### Putative signatures of selection

**Figure 2:**
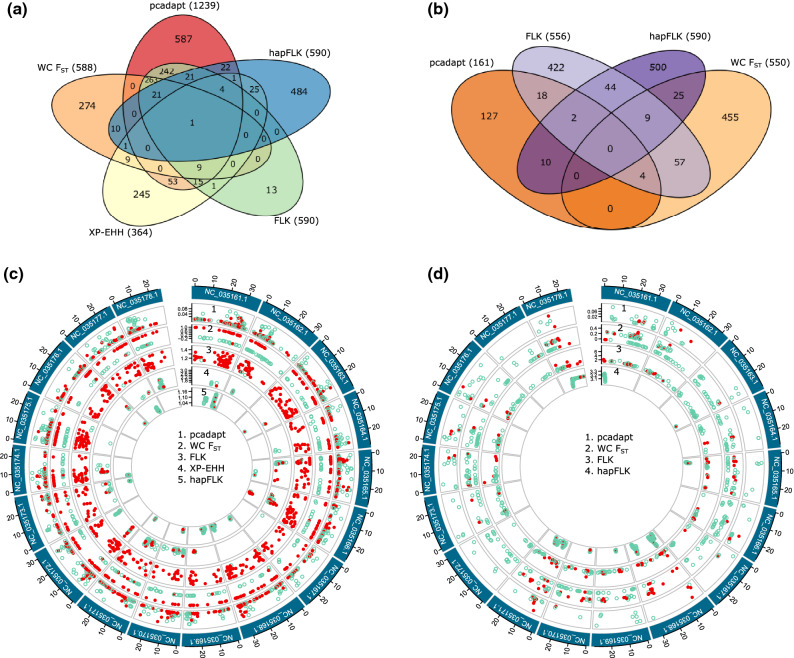
Summary of genome scans for signatures of selection considering different groups of manioc (*Manihot esculenta*) samples. Venn diagrams showing the number of outlier SNPs detected for each test (within parenthesis) and the overlap among them (numbers inside ellipses) for (**a**) wild and cultivated manioc, and (**b**) the groups of varieties per biome. The genomic context of outlier SNPs is illustrated in circular plots for (**c**) the groups of wild and cultivated manioc, and d) the groups of varieties per biome. Each manioc chromosome is represented by a different box (10 Mb tick sizes), and their names are coded according to the manioc genome *Manihot esculenta v6* (NCBI PRJNA234389). The outlier SNPs are represented by dots for each test, which are shown in different layers. The outlier SNPs detected by at least two tests are highlighted in red.

The tests for selective signatures contrasting the wild and cultivated manioc identified a total of 2301 outlier SNPs (*pcadapt*: 1239; *F*_*ST*_: 588; *FLK*: 590; *hapFLK*: 590; *XP-EHH*: 364), of which 698 SNPs were identified by at least two different methods (Fig. 2a,c). When contrasting the groups of varieties per biome, we identified a total of 1673 outlier SNPs (*pcadapt*: 161; *F*_*ST*_: 550; *FLK*: 556; *hapFLK*: 590), of which 169 SNPs were identified by at least two different methods (Fig. 2b,d). Only two outlier SNPs were common for both criteria, and we considered that 865 outlier SNP loci showed putative signatures either of the selection of cultivated manioc from the wild ancestor, or for diversification of manioc in different cultivation environments. A total of 5174 effects were predicted for these outlier SNPs (Supplementary Table S3), of which 569 were within introns and 534 within exons (269 synonymous mutations and 265 non-synonymous). These numbers are greater than the number of outlier SNPs because the effects were predicted for all the alternative transcripts of the genes.

Among the loci with putative selective signatures, 680 SNPs were in 663 different predicted manioc genes. These genes showed a total of 1176 annotations distributed in 33 different GO classes (Supplementary Fig. S2). The most frequent GO annotations were associated with the molecular functions of binding (215 genes) and catalytic activity (188 genes), and the biological process of metabolism (181 genes). Consistent with this, the most frequent enriched GO annotations were binding to ATP (58 genes) and biding to metal ion (49 genes) (Supplementary Table S4). A total of 69 manioc genes with outlier SNPs were similar to PRGdb 3.0 resistance genes (45 with identity > 90%) belonging to six different classes (according to their protein domains), and most of them (38) had a kinase domain. A total of 576 manioc genes with outlier SNPs were similar to Swiss-Prot proteins (306 with identity > 60%) related to many functions, including plant growth and development, organ sizes, root, flowering, and biotic and/or abiotic stresses. Many genes had similarities with proteins related to more general cellular processes, such as cell proliferation/elongation, transcription regulation, chloroplast activity, signaling, and ubiquitination. The variety of functions is exemplified by the descriptions for 21 of these proteins (Supplementary Material Appendix 1), that we used to guide our discussion. Supplementary Table S5 summarizes blastp results.

### Genome-wide diversity

The analyses below were performed considering the 10,917 putatively neutral SNPs that were not identified as outliers by more than one test of selection. We report the results based on all the 92 samples evaluated, but similar results were observed when considering only the varieties conserved in the gene bank (Supplementary Tables S6, S7 and S8). Likely due to the larger sampling number, cultivated manioc had greater allelic diversity, more private alleles, and higher expected heterozygosity than wild manioc (Table 1). Within cultivated manioc, the sweet varieties had greater observed than expected heterozygosity (*H*_*O*_ = 0.322, *H*_*E*_ = 0.308), while the bitter varieties had a slight deficit of heterozygotes (*H*_*O*_ = 0.295, *H*_*E*_ = 0.309), although its small positive inbreeding coefficient (*f* = 0.047) was not significant. The varieties from different biomes had similar levels of genetic diversity (Table 1). Amazonia was the only biome with a slight deficit of heterozygotes (*f* = 0.062), but it had the greatest number of private alleles (*PA* = 153).

**Table 1:** Estimates of genetic diversity and inbreeding based on 10,917 neutral SNPs for groups of manioc (*Manihot esculenta*) varieties.

Groups	N	*A*	*%P*	*PA*	*H*_*O*_ (95%CI)	*H*_*E*_ (95%CI)	*f* (95%CI)
Cultivated	84	21,516	98.5	7054	0.324 (0.321; 0.328)	0.315 (0.312; 0.318)	− 0.030 (− 0.071; 0.005)
Bitter	16	21,009	97.9	60	0.295 (0.291; 0.298)	0.309 (0.306; 0.312)	0.047 (− 0.014; 0.093)
Sweet	43	21,435	98.2	43	0.322 (0.318; 0.326)	0.308 (0.305; 0.310)	− 0.047 (− 0.105; − 0.001)
Wild	8	14,780	67.7	318	0.103 (0.098; 0.105)	0.122 (0.118; 0.125)	0.177 (− 0.040; 0.354)
**Biomes**							
Amazonia	22	21,240	98.8	153	0.293 (0.290; 0.296)	0.312 (0.310; 0.315)	0.062 (0.015; 0.097)
Cerrado	30	21,108	98.2	16	0.342 (0.337; 0.346)	0.298 (0.295; 0.301)	− 0.145 (− 0.216; − 0.090)
Atlantic Forest	27	21,142	99.7	7	0.325 (0.321; 0.329)	0.300 (0.297; 0.303)	− 0.085 (− 0.167; − 0.026)
Pantanal	5	19,604	92.7	0	0.349 (0.344; 0.354)	0.285 (0.281; 0.288)	− 0.226 (− 0.492; − 0.107)

The groups of cultivated maniocs, and each distinct biome include varieties for which there are no information about reputed toxicity (non-designated) in the gene bank passport data. *N* = number of samples, *A* = total number of alleles, *%P* = percentage of polymorphic loci, *PA* = number of private alleles, *H*_*O*_ = observed heterozygosity, *H*_*E*_ = expected heterozygosity, *f* = inbreeding coefficients, *95%CI* = 95% confidence intervals.

According to the AMOVAs, greater proportions of the genetic variance were found within groups (Table 2). As expected, the greatest divergence among groups was observed between wild and cultivated manioc (φ_ST_ = 0.32, *p* < 0.001), followed by the divergence between the groups of biomes when compared to wild manioc (φ_CT_ = 0.31, *p* < 0.001). The divergence among different biomes was small, yet significant (φ_ST_ = 0.03, *p* < 0.001). Pair-wise estimates of *F*_*ST*_ between cultivated and wild manioc were high (Table 3). The bitter varieties were slightly more divergent from wild manioc (*F*_*ST*_ = 0.357) than the sweet varieties (*F*_*ST*_ = 0.344), and the divergence between bitter and sweet varieties was much lower (*F*_*ST*_ = 0.045), yet significant. All the biomes were highly divergent from wild manioc, with *F*_*ST*_ ranging from 0.34 (Amazonia) to 0.43 (Pantanal). The divergence between biomes ranged from non-existent (Atlantic Forest vs. Pantanal = − 0.015) to moderate (Amazonia vs. Cerrado = 0.065). Amazonia had the greater and significant estimates of divergence in relation to the other biomes, although *F*_*ST*_ was low to moderate (Table 3).

**Table 2:** Analysis of molecular variance based on 10,917 neutral SNPs, showing the genetic variation within and among hierarchical groups of manioc (*Manihot esculenta*) varieties.

Source of variation	DF	Sum of squares	Variance components	Percentage of variance	φ-statistics
Between Wild and Cultivated	1	22,823.83	729.25	32.5	φ_ST_ = 0.32 (*p* < 0.001)
Within Wild and Cultivated	182	276,088.28	1516.97	67.5	
Total	183	298,912.11	2246.22		
Among Bitter, Sweet, and Wild	2	26,517.04	341.13	19.1	φ_ST_ = 0.19 (*p* < 0.001)
Within Bitter, Sweet, and Wild	131	189,459.88	1446.26	80.9	
Total	133	215,976.92	1787.39		
Among Biomes	3	11,073.90	54.76	3.4	φ_ST_ = 0.03 (*p* < 0.001)
Within Biomes	164	254,820.13	1553.78	96.6	
Total	167	265,894.03	1608.54		
Between Biomes and Wild	1	22,823.83	693.60	31.1	φ_ST_ = 0.34 (*p* < 0.001)
Among groups within Biomes and Wild	3	11,073.90	56.64	2.5	φ_SC_ = 0.03 (*p* < 0.001)
Within Biomes and Wild	179	265,014.38	1480.53	66.4	φ_CT_ = 0.31 (p = 0.19)
Total	183	298,912.11	2230.77		

All the analyses, except among bitter, sweet, and wild, include varieties for which there are no information about reputed toxicity (non-designated) in the gene bank passport data. DF = Degrees of freedom.

**Table 3:** Pairwise estimates of genetic divergence (Weir & Cockerham’s *F*_*ST*_ (1984)) among groups of manioc (*Manihot esculenta*) varieties, based on 10,917 neutral SNPs.

Groups	Bitter	Sweet	Biomes	Amazonia	Cerrado	Atlantic Forest	Pantanal
Sweet	0.045*		Cerrado	0.065*			
Wild	0.357*	0.344*	Atlantic Forest	0.045*	0.017		
			Pantanal	0.044*	− 0.025	− 0.015	
			Wild	0.342*	0.365*	0.362*	0.433*

Each biome also includes varieties for which there are no information about reputed toxicity (non-designated) in the gene bank passport data.

*Significant estimates at *p* < 0.01.

**Figure 3:**
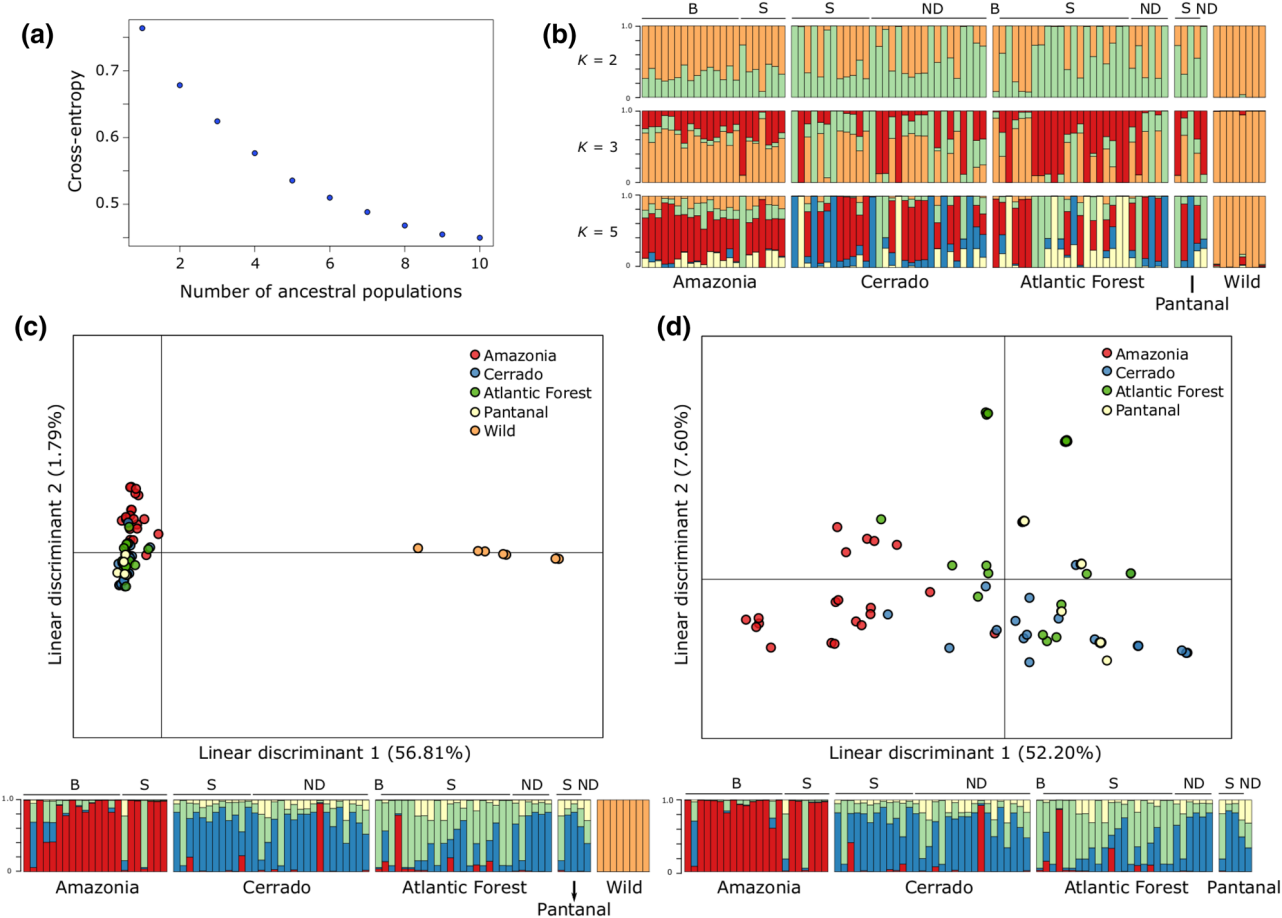
Genetic structure of 92 manioc (*Manihot esculenta*) varieties based on 10,917 neutral SNPs. (**a**) Plot of cross-entropy estimates for different numbers of ancestral populations (*K*) in sparse non-negative matrix factorization (sNMF) showing no evident flatting point in the curve corresponding to the most-likely number of ancestral populations. (**b**) Bar plots of sNMF ancestry coefficients for *K* = 2, 3, and 5. Discriminant analyses of principal components (DAPC) considering: (**c**) the groups of wild manioc and the different biomes, and (**d**) only the cultivated varieties grouped by biomes. The respective membership coefficients of each DAPC are shown as bar plots below scatter plots. Cultivated manioc is ordered in the sNMF and DAPC bar plots according to the biomes and their reputed toxicity (B = bitter, S = sweet, ND = non-designated).

The high divergence between wild and cultivated manioc, and the low divergence among biomes were also evident in sNMF and DAPC (Fig. 3). There was no flatting point in the curve of cross-entropy estimates in sNMF, suggesting the absence of major genetic structure (Fig. 3a). Therefore, we evaluated the correspondence of the ancestry coefficients for *K* = 2, 3 and 5 with the respective groups of cultivated vs. wild, bitter vs. sweet vs. wild, and the four biomes vs. wild (Fig. 3b). The most evident genetic structure was observed between the wild and cultivated manioc, with high admixture between the groups of bitter and sweet varieties or among biomes. DAPC results were similar, showing high divergence between wild and cultivated manioc, and a great overlap of varieties from different biomes (Fig. 3c,d). Besides the great genetic admixture, the DAPC plots also identify some highly divergent varieties from different biomes. Because DAPC maximizes between-group variations, Amazonia was somewhat more divergent in relation to the other biomes, just as suggested by the pairwise *F*_*ST*_ estimates.

## Discussion

There are many methods for the detection of selective signatures based on the significant deviation of outlier markers from the distribution of a given statistic measured under a specific model^53^. However, deviance from model assumptions and covariance with sampling strategy, demography, underlying genetic structure, and other specific factors may lead to the detection of false positives^53–56^. A general approach to account for these limitations is to combine the results of different outlier tests^57,58^. The variable number of outlier SNPs detected by the tests we performed reflect their different underlying models. We discuss below the possible biological significance of some outlier SNPs consistently identified by different tests.

The reduction of genetic diversity associated with domestication bottlenecks, or resulting from multiple founding effects during crops’ dispersals^59,60^, might have affected plant defense and resistance mechanisms^61^. The presence of outlier SNPs in putative resistance genes from different classes suggests that the gene bank conserves important genetic resources for the crop. For example, the outlier SNPs *Pst_1761* and *Pst_6563* were identified in genes similar to the disease resistance proteins RPS2 and RPM1, which are involved in the response to bacterial blight. In manioc, “cassava bacterial blight” is caused by *Xanthomonas campestris* pv. *manihotis* (or *X. axonopodis* pv. *manihotis*) that is found in cultivation areas throughout the world and is one of the most serious diseases affecting the crop^11,62^. We also identified outlier SNPs (*Nsi_172* and *Pst_4209*) in genes similar to proteins involved in resistance to powdery mildew fungus, which in manioc is known as ash disease and is caused by *Oidium manihotis* Henn^62^. Although ash disease is widespread, it is not considered of great importance due to its superficial lesions^62^. Agronomic trials would be important to confirm if some of the varieties conserved in the gene bank may be sources of resistance alleles for these diseases. Pest and disease resistance may be the principal weakness of manioc to adapt to climate change^63^.

Most of the putative manioc resistance genes had other functions probably due to their catalytic kinase domains, which play many key roles in eukaryotes^64^ including signaling and plant defense responses^65,66^. Other outlier SNPs were in genes related to general cellular processes, such as ubiquitination (*Nsi_2393*, *Nsi_3361* and *Pst_4290*) and transcriptional regulation (*Nsi_3361*, *Pst_8996*, *Pst_9853* and *Pst_9959*). These processes are central in the expression and regulation of several other genes, and therefore are likely to be involved in adaptations, including domestication traits^67,68^.

Outlier SNPs in genes putatively involved with development may be genetic signatures of manioc domestication and selection for different traits of interest in response to distinct human preferences. Manioc was domesticated for its starchy roots, and we identified SNPs in genes putatively involved in root formation (*Nsi_2393*, *Nsi_3361*, *Pst_4290*, *Pst_8996* and *Pst_9959*), organ size (*Nsi_3361*) and shape (*Pst_1010*), and in starch metabolism (*Pst_5701*). These SNPs may be of agronomic importance because the current major objectives of breeding for food and industrial uses include increasing the root/stem ratio, improving starch quality, and increasing starch content in roots^69,70^. We also found outlier SNPs in genes putatively involved in the development of shoots (*Nsi_3361 and Nsi_3540*) and branching (*Pst_6259*). Domestication often resulted in changes in shoot architecture that facilitate plant growth and harvesting^71,72^. Contrasting with the highly branched habit of ssp. *flabellifolia*^21^, farmers prefer sparsely branched manioc plants because they may provide thicker stem cuttings^73^. Some outlier SNPs were in genes putatively involved in flower development (*Pst_7007*, *Pst_8971*, *Pst_9853* and *Pst_9959*), fertilization (*Nsi_172* and *Pst_4209*), and gametogenesis (*Nsi_3361*). These selective signatures may result from the relaxation of selective pressures on sexual fertility caused by domestication for vegetative propagation^74^. Flowering is variable in manioc and about 60% of the pollen produced may remain viable, although manioc cultivars commonly have male sterility and low seed/flower ratios^11^. We also identified outlier SNPs putatively involved in cotyledon development (*Nsi_2289* and *Nsi_4509*); this was in a manioc gene similar to *Arabidopsis thaliana* STY46, a protein kinase involved in chloroplast biogenesis and differentiation in cotyledons^75^. The seedlings of domesticated and wild manioc have contrasting functional differences^76^. The cotyledons are hypogeal in the seedlings of ssp. *flabellifolia*, providing additional opportunities for plant regrowth after damage. In domesticated manioc, cotyledons are epigeal, foliaceus, and photosynthetically active, collaborating with rapid plant growth^76^.

Growth resilience under distinct environments was essential for the spread and adaptation of crops to regions outside their domestication centers^71^. The outlier SNP *Pst_29* was in a gene putatively involved in the metabolism of glycosinolates, secondary compounds that may act in plant defense to a wide range of enemies^77^. Ecological shifts to resource-richer cultivation environments may have relaxed the selection for chemical and physical defenses in some crops^24^. For manioc, natural selection might have been important to maintain defense mechanisms because cultivation in anthropic landscapes made plants more apparent to pests and herbivores^24^. This is especially important in the context of active human selection for low toxic sweet manioc and cultivation in herbivore and pathogen-rich tropical regions^24^, such as Amazonia and other Brazilian biomes. We also found outlier SNPs (*Nsi_172*, *Pst_1010*, *Pst_3333, Pst_4209* and *Pst_5985*) possibly involved in cell proliferation and elongation. Genes controlling cell division are frequently associated with domestication and diversification processes because they often resulted in increased organ sizes^78,79^. The roots of cultivated manioc have greater amounts and larger starch granules, but not larger cell sizes, than the wild relatives^11,80^. However, the cessation of cell division and expansion may play an important role during manioc response to drought stress^81,82^. Another outlier SNP (*Pst_7007*) was in a gene that may act in responses to salt, drought, and cold stresses. Responses to abiotic stresses were important crop adaptations for the ecological shifts from the wild to the cultivation environment and when they started to be dispersed^24,71^. In the specific case of manioc, abiotic resistance is relevant to crop adaptability to marginal areas and might be key for manioc adaption to harsher future climates^63^.

We recognize that the type of genomic library and the limited sampling number of some groups of varieties may have introduced bias in the analyses^83,84^. Although the groups of manioc varieties are not true populations, some aspects, such as the possibility of crossings and incorporation of sexual plants into clonally propagated varieties, make these groups biologically meaningful. Moreover, genetic approaches provided interesting results even when using generic groups of manioc varieties^85,86^ and limited sample sizes in other study systems^87,88^. The selective signatures discussed above should be regarded as initial hypotheses and further genome-wide association studies and quantitative trait loci mapping are required to confirm their biological significance^71,89^. Nonetheless, this new information may guide future bottom-up approaches to characterize the genomic changes and their associated phenotypic effects^90^ relevant to manioc evolution under domestication and to assist breeding strategies, contributing to our understanding about adaptive genes in crops.

The genetic diversity of varieties from different biomes (*H*_*E*_ ranges from 0.285 to 0.312) was similar to that observed in previous studies and suggests that the gene bank conserves high levels of genetic diversity, despite its limited size. Within Embrapa Brazilian manioc germplasm bank, Albuquerque et al.^49^ reported an overall *H*_*E*_ = 0.29 (biallelic SNPs) and Ogbonna et al.^91^ found *H*_*O*_ ranging from 0.26 to 0.39 across genetic groups. Similar results were observed for the gene banks of the International Institute of Tropical Agriculture (IITA, *H*_*E*_ = 0.334) and the International Center for Tropical Agriculture (CIAT, *H*_*E*_ = 0.341)^43^. This high genetic diversity is explained by the complex evolutionary dynamics of the crop under traditional cultivation, that results in varieties consisting of a predominant clone plus individuals morphologically similar, but genetically distinct^28^. Although smallholder farmers propagate manioc exclusively by stem cuttings, crossings between different varieties may occur producing sexual seeds that become part of the soil seed bank and may sprout amid clonally propagated plants^27,92^. After harvesting, the farmers may incorporate stem cuttings of these sexual plants into their clonal stocks leading to the amplification and maintenance of genetic diversity in the local scale^30^. The high genetic diversity observed in the different biomes may be explained by the widespread occurrence of the incorporation of sexual plants to clonal varieties, as well as by the selection for distinct human preferences under diverse ecological and cultural contexts^68,93^. Genetic evidence for different human preferences in distinct ecogeographic contexts has already been reported in different regions of South America for manioc^20,94^ and other crops^95,96^.

The crop’s reproductive biology and other traditional farming practices may explain the low to moderate genetic divergence among biomes (*F*_*ST*_ ranging from − 0.025 to 0.065) and the high admixture observed in clustering analyses (Fig. 3). Exchange networks of manioc varieties have been reported at local and ample geographic scales^35,48^. These exchange networks facilitate geneflow between distinct varieties at local and broader geographical scales. Genetic admixture among varieties from distinct geographical locations are commonly associated with extensive exchange networks of manioc and other crops^36,97,98^. This low overall genetic divergence may also reflect the initial common dispersal of landraces from their domestication center in Amazonia^20,99^. The Amazonian varieties were somewhat more divergent in relation to the other biomes (Fig. 3d, Table 3), possibly because almost all bitter varieties were from this region. The influence of ecogeographic variation in the distribution of genetic diversity in manioc is variable^20,91,100^, but the existence of divergent varieties from different biomes may also reflect the selection under distinct ecological and cultural contexts. Nonetheless, the genetic divergence between bitter and sweet manioc seems to be higher in Amazonia than outside this region^85,86^. It is possible that divergent selective pressures for bitterness or sweetness are more relaxed outside Amazonia, because the crop’s dispersals were not always accompanied by cultural appropriation^13^. Knowledge about adequate processing to avoid intoxication after the consumption of bitter manioc landraces is essential to achieve food security where people rely on manioc cultivation^39^.

The remarkable divergence between wild and cultivated manioc was expected given the long history of the crop diversification under human selection and cultivation^16^. This result may also reflect many founding events^59^ that accompanied the rapid spread of the crop across the Neotropics^23^ and the wide dispersal across the world. The observed genetic divergence was similar to our previous study^85^, but more geographically extensive sampling of wild populations would improve our understanding about the current population dynamics between wild and cultivated manioc. Recent genomic approaches evidenced introgressions from some wild relatives in the genome of cultivated manioc^44,45^. Because the primary gene pool of manioc consists of 13 species^101^, introgressions may have been contributing to the extant genetic diversity of the crop. Therefore, genome-wide studies in cultivated manioc and different wild *Manihot* species could greatly contribute to our understanding about the evolution of the crop.

In this study, we observed high levels of genome-wide diversity in manioc varieties from different Brazilian biomes. It is noteworthy that the genetic diversity also included putative adaptive variation, which may be associated with the crop’s domestication and its cultivation in distinct environmental contexts with different human preferences. Some of the signatures of selection may be associated with resistance genes and agronomic traits of interest, which might have practical importance for breeding purposes. The varieties conserved in the gene bank can be used as sources for reintroductions into smallholder communities^102^ : the highly genetic divergent varieties are important resources for their specific regions of origin, while the admixed varieties may adapt well to cultivation in various locations. This study reinforces the importance of ex situ collections for the conservation of the crops’ genetic resources, although it is financially and technically challenging to maintain active gene banks^8,103^. Our study also highlights the necessity of maintaining traditional practices of cultivation, since they are often associated with the management of a great diversity of other native crops^102,104,105^. In the context of a changing world, the characterization and conservation of agrobiodiversity is essential for the appropriate management of their genetic resources and ultimately for food security, especially of poor people.

## Methods

### Plant material and DNA isolation

We sampled apical leaves of the 78 manioc varieties (1 plant/accession) from the gene bank at the Luiz de Queiroz College of Agriculture, University of São Paulo, Piracicaba, São Paulo, Brazil (22° 42′ 26.8″ S; 47° 38′ 17.8″ W). These varieties were originally collected in smallholder farmer communities in six Brazilian states, located in four different biomes: Amazonia (16), Cerrado (30), Atlantic Forest (27) and Pantanal (5) (Fig. 1, Supplementary Table S1). Of these 78 cultivated varieties, 40 were originally recognized by the farmers as sweet varieties, 13 as bitter varieties, but this information was unavailable for 25 varieties, which are identified in Fig. 1 and Supplementary Table S1 as ND (non-designated). We also evaluated other samples collected in a previous study^85^: eight *M. esculenta* ssp. *flabellifolia* samples collected in the center of manioc domestication in Rondônia state, and six Amazonian varieties (three bitter and three sweet). We used these samples as references for the crop’s closest wild relative, and for the major groups of cultivated manioc, respectively. The collections of these samples were registered in the Brazilian National Council for Genetic Patrimony CGEN (numbers A7994B4 and AEA71DE), according to Brazilian Law 13123 (20 May 2015). These registers also characterize our study as basic scientific research, enabling the experiments that we performed. The plant materials and methods employed in the present study are in compliance with local and national regulations. Because the manioc varieties are conserved in vivo in the gene bank, they were not deposited in herbarium, however DNA vouchers are stored in our laboratory. In addition, because all manioc varieties have been cultivated for a long time in local communities of smallholder farmers, no formal taxonomic identification was performed at the time of sampling to establish the gene bank.

The leaves were dehydrated with silica gel in paper bags and stored at -20 °C. We obtained total genomic DNA from 50 mg of leaf samples following the protocol described by Doyle and Doyle^106^ and inspected DNA quality with electrophoresis in agarose 1% (w/v) gels stained with ethidium bromide. We estimated DNA concentrations with dsDNA BR Assay quantification kit for Qubit3 fluorometer (Invitrogen), and normalized DNA concentrations to 25 ng∙μL^-1^.

### Genomic libraries and SNP identification

We prepared two double-digest genotyping-by-sequencing (ddGBS) libraries as described by Poland et al.^107^. Briefly, 175 ng of genomic DNA were digested with *PstI* and *MseI* for one library, and *NsiI* and *MspI* for the other (all enzymes from New England Biosciences). The restriction fragments were ligated to adaptors complimentary to each of the restriction sites (including 96-plex *PstI* or *NsiI* adaptor sets with unique 4–9 bp barcode sequences). Ligation products of each sample were pooled and enriched for fragments containing different adapters in both ends through PCR. The concentrations of the ddGBS libraries were estimated using the NEBNext Library Quant kit for Illumina (New England Biosciences) through real-time PCR, and the libraries’ profiles were inspected using the DNA 12000 Analysis kit for Bioanalyzer 2100 (Agilent). Each ddGBS library was sequenced twice in the NextSeq550 (Illumina) platform (single-end, 150 bp).

We inspected the quality of raw reads using FastQC^108^, and due to a large amount of 3’-end adaptors we trimmed the reads to 100 and 80 bp for *PstI*-*MseI* and *NsiI*-*MspI* libraries, respectively. We performed read trimming and demultiplex according to specific barcodes using the module *process_radtags* of Stacks 1.42^109^. We aligned the demultiplexed reads against the manioc genome *Manihot esculenta v6* (NCBI PRJNA234389)^47^ using Bowtie 2.2.1^110^ with the “end‐to‐end” and “sensitive” configurations. We identified SNPs separately for each library using SAMtools 0.1.19^111,112^ and VCFtools 0.1.17^113^, retaining only biallelic markers and only one SNP per read to avoid explicit linkage. Candidate SNPs had sequence depth ≥ 5X, minor allele frequency ≥ 0.05, mapping quality ≥ 13 and were present in at least 90% of the samples. The SNPs identified in each library were merged using MergeVcfs from Picard (http://broadinstitute.github.io/picard). Then, we used VCFtools 0.1.17^113^ and SAMtools 0.1.19^111^ to filter out SNPs within 100 bp and within windows of 1000 SNP sites with linkage disequilibrium (LD) r^2^ ≥ 0.8, and to retain the SNPs observed in *Manihot esculenta v6* chromosomes.

### Genome scans

We used five different methods (*pcadapt*, *F*_*ST*_, *FLK*, *hapFLK*, and *XP-EHH*) with distinct underlying models to detect putative selective signatures (outlier SNPs). These analyses were performed considering two scenarios: i) the groups of wild versus cultivated manioc (including sweet, bitter, and non-designated varieties), and ii) the groups of varieties per biomes (without wild samples, but with non-designated varieties). With this design we aimed to identify loci with putative selective signatures related to either the domestication of manioc or the diversification of the crop in different cultivation environments.

Pcadapt is based on a principal component analysis (PCA) without assuming any explicit genetic model^114^. The detected outlier SNPs are associated with the first *K* principal components with greater contribution to the observed genetic structure. We used pcadapt 4.03^114^ for R^115^ to test 1 to 20 *K* principal components. We detected outliers based on *K* = 2 for wild versus cultivated manioc, and *K* = 5 for the biomes (Supplementary Fig. S3). The estimation of genetic differentiation among populations, measured by *F*_*ST*_ or related statistics, is a classic method for detecting selective signatures that reflect a broad range of scenarios, such as selection of standing variation or incomplete sweeps^116^. We estimated Weir and Cockerham’s *F*_*ST*_^117^ among the groups of samples for each locus using diveRsity^118^ for R^115^. *FLK* is similar to *F*_*ST*_, but it accounts for hierarchical structures using a kinship matrix to model the covariance of the populations’ allele frequencies^119^. We estimated *FLK* for each locus using hapFLK 1.4 (https://forge-dga.jouy.inra.fr/projects/hapflk) based on the calculation of Reynold’s^120^ distances from our data.

The three methods above are based on individual markers, and we also applied two tests (*hapFLK* and *XP-EHH*) considering “long-range haplotypes” that account for variation in recombination rates by comparing haplotypes to other alleles in adjacent loci^121^. *HapFLK* was built upon *FLK* for the detection of positive selection, including incomplete sweeps, from multiple populations and is robust in the presence of bottlenecks and migration^116^. Complimentarily, the cross-population extended haplotype homozygosity (*XP-EHH*) detects loci that have swept to near fixation (hard sweeps) within a specific population^121^. The hard sweeps detected based on XP-EHH estimates assume one ancestral and one derived population. While this makes sense in the case of the groups of wild and cultivated manioc, it might not be straightforward when contrasting different biomes. Even if we considered manioc varieties from Amazonia as ancestral to the varieties from the other biomes, we would follow a different criterion (pairwise comparisons among biomes) in relation to the other tests performed for the identification of selective signatures that considered the overall genetic divergence between biomes. For that reason, this analysis was performed only considering the groups of cultivated and wild manioc. For these two analyses, we used fastPHASE 1.4^122^ to estimate missing data and reconstruct haplotypes. We used a perl script (https://github.com/lstevison/vcf-conversion-tools) to convert VCF files to fastPHASE input format and then performed 20 runs of the expectation–maximization (EM) algorithm with default configurations. We estimated *hapFLK* simultaneously with *FLK* considering the same Reynold’s distance matrix and using 15 EM runs. We estimated *XP-EHH* using the functions *scan_hh()*, *ies2xpehh()* and *calc_candidate_regions()* in rehh 2.0^123^ for R^115^. We set the functions to use unpolarized data, and to consider the XP-EHH estimates in the direction of wild to cultivated manioc (positive selection of alleles in the cultivated samples).

We considered as outlier SNPs the loci with *q*‐values ≤ 0.10 in *pcadapt*, loci at the top and bottom 2.5% of the *F*_*ST*_ estimates (reflecting positive and balancing selection, respectively), and the loci at the top 5% of the *FLK*, *hapFLK* and *XP-EHH* estimates. Then, we considered as loci putatively under selection those SNPs that were detected in at least two of the five (four) methods used for the groups of wild versus cultivated manioc (groups of varieties per biome).

We evaluated the predicted effects of outlier SNPs using SnpEff^124^. We recovered the Gene Ontology (GO) annotations, which summarize the information about biological processes, molecular functions, and cellular components^125^ of the gene sequences with outlier SNPs extracted from *Manihot esculenta v6* with BEDTools 2.30.0^126^. Genes with outlier SNPs were tested for enrichment of gene function descriptions using topGO 2.44.0^127^ for R^115^ based on default configurations and a threshold of *p* < 0.01 for Fisher’s exact tests. We compared the amino acid sequences of the predicted manioc genes with i) the predicted protein sequences of 2609 manioc R-genes from the Plant Resistance Gene database (PRGdb 3.0)^128^; and ii) the Swiss-Prot database from UniProt (www.uniprot.org) using blastp from blast 2.7.1^129^. Then, we further assessed putative functional annotations described in UniProt for the blastp hits with the arbitrary threshold of identity ≥ 90% (PRGdb) or ≥ 60% (Swiss-Prot).

### Population genetics analyses

We performed the analyses of genetic diversity and structure considering only the putatively neutral SNPs (those that were not detected as outliers by at least two of the tests described above). We estimated the genetic diversity [total number of alleles (*A*), percentage of polymorphic loci (*%P*), number of private alleles (*PA*), observed (*H*_*O*_) and expected (*H*_*E*_) heterozygosities] and the inbreeding coefficients (*f*) using diveRsity^118^ and PopGenKit^130^ for R^115^. *H*_*O*_, *H*_*E*_, and *f* confidence intervals were obtained with 1000 bootstraps.

We evaluated the genetic variation within and among groups of varieties with analyses of molecular variance (AMOVA), estimated pairwise genetic divergence (F_ST_) among groups, and obtained their associated significance based on 20,000 permutations using Arlequin 3.5^131^. We investigated the patterns of genetic structure using sparse non-negative matrix factorization (sNMF)^132^ and discriminant analysis of principal components (DAPC)^133^. sNMF assumes that the genetic data originate from the admixture of *K* unknown parental populations and estimates ancestry coefficients from multilocus genotypes^132^. This analysis is similar to other model-based approaches, such as Structure^134^, with the advantage of being robust to departures from traditional population genetic model assumptions^132^. We tested from 1 to 10 *K* ancestral populations with 200,000 iterations, ten repetitions for each *K* value, and used the cross-entropy criterion to visualize the results of the simulations. We performed this analysis using LEA^135^ for R^115^. Complimentarily, DAPC summarizes information from large datasets (like genotypes from thousands of SNPs) to assign individuals to clusters without a pre-defined genetic model^133^. DAPC is useful for the assessment of genetic structure based on SNP datasets because it maximizes the variation among groups while minimizing the correlations between the original variables (such as LD)^133^. We performed DAPCs in adegenet^136^ for R^115^ based on the groups of wild and cultivated manioc from different biomes, and only for the groups of varieties per biomes (without wild samples). We opted to use these groups in DAPC because sNMF was performed without any a priori classification and suggested no clear number of ancestral populations (see “Results”). We used the function *optim.a.score()* to apply the alpha-score optimization to obtain the number of principal components (PCs) that reduced over-fitting of DAPCs membership coefficients^136^. After this procedure we retained nine PCs in the analysis considering the four biomes plus wild manioc, and 11 PCs in the DAPC considering only the four biomes.

## Supplementary Information

Supplementary Information 1.

Supplementary Information 2.

## Data Availability

Final SNP data uploaded as online Supplementary Table S2. Sequence alignments (bam files) were deposited in NCBI SRA (library *Nsi*I + *Msp*I: PRJNA748763, library *Pst*I + *Mse*I: PRJNA748779).
