# Supplementary material for: Selective signatures and high genome-wide diversity in traditional Brazilian manioc (Manihot esculenta Crantz) varieties
Source: Sci Rep. 2022 Jan 24;12:1268. doi: 10.1038/s41598-022-05160-8 (PMC8786832; doi:10.1038/s41598-022-05160-8)
Supplement: Supplementary file 1 — Supplementary Information 1. [file 41598_2022_5160_MOESM1_ESM.docx]

**Selective signatures and high genome-wide diversity in traditional Brazilian manioc (*Manihot esculenta* Crantz) varieties**

Alessandro Alves-Pereira^1,2^, Maria Imaculada Zucchi^3^, Charles Roland Clement^4^, João Paulo Gomes Viana^5^, José Baldin Pinheiro^6^, Elizabeth Ann Veasey^6^, Anete Pereira de Souza^1,2*^

1 Departamento de Biologia Vegetal, Instituto de Biologia, Universidade Estadual de Campinas (UNICAMP). Av. Cândido Rondon, 400, Cidade Universitária, 13083‐875, CP: 6010, Campinas, SP, Brazil.

2 Centro de Biologia Molecular e Engenharia Genética, Universidade Estadual de Campinas (UNICAMP). Av. Cândido Rondon, 400, Cidade Universitária, 13083‐875, CP: 6010, Campinas, SP, Brazil.

3 Agência Paulista de Tecnologia dos Agronegócios (APTA), Pólo Centro-Sul. Rodovia SP 127, km 30, 13400-970, Piracicaba, SP, Brazil.

4 Instituto Nacional de Pesquisas da Amazônia (INPA). Av. André Araújo, 2936, Petrópolis, 69067-375, Manaus, AM, Brazil.

5 Department of Crop Sciences, University of Illinois at Urbana-Champaign (UIUC). AW-101 Turner Hall, 1102 South Goodwin Avenue, 61801-4798, Urbana, IL, USA.

6 Departamento de Genética, Escola Superior de Agricultura “Luiz de Queiróz”, Universidade de São Paulo (ESALQ/USP). Av. Pádua Dias, 11, 13400-970, Piracicaba, SP, Brazil.

*Corresponding author: Anete Pereira de Souza. E-mail: [anete@unicamp.br](mailto:anete@unicamp.br), Phone number: +55-19-3521-1132

**Supplementary Material**

**Appendix 1** Predicted effects and annotations of 21 SNP markers with putative selective signatures considering different groups of manioc (*Manihot esculenta*) samples.

| SNP ID ^a^ | Genomic location ^b^ | Gene name ^c^ | Outlier tests ^d^ | Predicted effect ^e^ | Name of the protein with Blastp similarity | Identity (%) | E-value ^f^ | Swiss-Prot Accession | Functional description ^g^ | Keywords |
| --- | --- | --- | --- | --- | --- | --- | --- | --- | --- | --- |
| Pst_1761 (G > T) | NC_035163.1 (2920331) | LOC110610533 (Manes.03G036300)* | G (FLK, pcadapt, FST) | Missense variant [280] Ala > Ser | Disease resistance protein RPS2 | 100 | 0 | Q42484 | RPS2 is a disease resistance protein involved in the response to *Pseudomonas syringae* ^1^. | Resistance to pathogens |
| Pst_6563 (C > T) | NC_035172.1 (1844961) | LOC110628374 (Manes.12G024600)* | G (FLK, pcadapt) | Missense variant [604] Thr > Ile | Disease resistance protein RPM1 | 100 | 0 | Q39214 | RPM1 is a disease resistance protein involved in the response to *Pseudomonas syringae* ^2^. | Resistance to pathogens |
| Nsi_172 (A > C) | NC_035161.1 (22495119) | LOC110630587 (Manes.01G102500)* | B (FLK, FST) | Synonymous variant [736] Leu > Leu | Receptor-like protein kinase FERONIA | 100 | 0 | Q9SCZ4 | FERONIA is a receptor-like protein kinase involved in growth cessation of compatible pollen tubes ensuring reproductive isolation barriers ^3^. Required for cell elongation during vegetative growth ^4^. Mediates sensitivity to powdery mildew ^5^. | Fertilization, Cell elongation, Powdery mildew |
| Pst_4209 (A > G) | NC_035167.1 (4576622) | LOC110618886 (Manes.07G046400)* | G (FLK, pcadapt) | Missense variant [440] Val > Ala | Receptor-like protein kinase FERONIA | 100 | 0 | Q9SCZ4 | Idem. | Idem. |
| Pst_3333 (G > A) | NC_035165.1 (20713381) | LOC110616124 (Manes.05G145600)* | G (hapFLK, pcadapt) | 5' UTR variant | Receptor protein kinase CLAVATA1 | 100 | 0 | Q9SYQ8 | CLAVATA1 acts in a signal transduction pathway controlling the balance between meristem cell proliferation and differentiation ^6^. | Development, Cell proliferation, Growth |
| Pst_1010 (C > T) | NC_035162.1 (1649693) | LOC110609084 (Manes.02G020500)* | G (FLK, pcadapt) | Missense variant [318] Ala > Thr | Mitogen-activated protein kinase kinase kinase YODA (YDA) | 99.4 | 0 | Q9CAD5 | YDA is involved in the first cell fate decisions in the zygote and the early embryo. It is involved in regulating coordinated local cell proliferation, which shapes the morphology of plant organs ^7^. | Cell proliferation, Organ shape |
| Pst_6259 (C > G) | NC_035171.1 (14640434) | LOC110626307 (Manes.11G094000)* | B (hapFLK, FST) | Synonymous variant [604] Gly > Gly | Proline-rich receptor-like protein kinase PERK12 (PERK12) | 100 | 0 | Q9ZUE0 | PERK12 regulates the auxin-related MAX (More Axillary Growth) pathway during shoot branching ^8^. | Branching |
| Nsi_4509 (A > T) | NC_035178.1 (777460) | LOC110606622 (Manes.18G008800)* | G (FLK, pcadapt) | Upstream gene variant | Serine/threonine-protein kinase (STY46) | 100 | 0 | F4JTP5 | SYT46 may be part of a cytosolic regulatory network involved in chloroplast protein import. Plays a role in chloroplast biogenesis and differentiation in cotyledons ^9,10^. | Chloroplast biogenesis and differentiation in cotyledons |
| Nsi_3361 (G > A) | NC_035173.1 (4723844) | LOC110630205 (Manes.13G043200) | B (FLK, FST) | Synonymous variant [148] Leu > Leu | 26S proteasome regulatory subunit 4 homolog A (RPT2A) | 96.4 | 0 | Q9SZD4 | RPT2A acts in degradation of ubiquitinated proteins and is required for the maintenance of postembryonic root and shoot meristems ^11^. It is involved in the regulation of organ size ^12^, in the regulation of gametogenesis ^13^, in the regulation of transcriptional and post-transcriptional gene silencing ^14^, tolerance to zinc deficiency ^15^, and fungal resistance ^16^. | Development (root and shoot), Organ size, Defense, Fungal resistance, Gene silencing, Gametogenesis, Ubiquitin |
| Pst_4290 (G > C) | NC_035167.1 (19228818) | LOC110618716 (Manes.07G083000) | G (FLK, pcadapt, FST) | Intron variant | Ubiquitin C-terminal hydrolase 12 (UBP12) | 85.6 | 0 | Q9FPT1 | UBP12 is involved in the processing of ubiquitinated proteins acting as positive regulator of root meristem development ^17^. | Root development, Ubiquitin |
| Nsi_2393 (C > A) | NC_035169.1 (5588020) | LOC110622644 (Manes.09G041200) | G (FLK, pcadapt, FST) | Intron variant | Ubiquitin C-terminal hydrolase 12 (UBP12) | 87.3 | 0 | Q9FPT1 | Idem. | Root development, Ubiquitin |
| Pst_8996 (A > C) | NC_035176.1 (24796226) | LOC110603073 (Manes.16G091200) | G (FLK, pcadapt, FST) | Synonymous variant [3] Val > Val | Auxin response factor 19 (ARF19) | 83.2 | 0 | Q8RYC8 | Auxin response factors (ARFs) are transcriptional factors. ARF19 may regulate lateral root formation ^18^. | Transcriptional factor, Root formation |
| Pst_5985 (G > C) | NC_035171.1 (262333) | LOC110626926 (Manes.11G002200) | G (FLK, pcadapt, FST) | 3' UTR variant | Glycosyltransferase-like KOBITO 1 (ELD1) | 77.1 | 0 | Q9C9Z9 | ELD1 is involved in the coordination between cell elongation and cellulose synthesis ^19^, in abscisic acid (ABA) and sugar responses essential for growth ^20^, maintaining normal organogenesis. It is involved with cell elongation and maintenance of root meristem identity ^21^. Acts as negative factor in light inhibition of hypocotyl elongation ^19^. | Growth, Cell elongation |
| Pst_5701 (C > T) | NC_035170.1 (5198647) | LOC110624912 (Manes.10G051700) | G (FLK, hapFLK, pcadapt, FST) | Intron variant | Pullulanase 1, chloroplastic (PU1) | 72.4 | 0 | Q8GTR4 | PU1 is involved in starch degradation and probably in starch synthesis ^22^. | Starch |
| Pst_7007 (C > T) | NC_035173.1 (1033679) | LOC110630343 (Manes.13G010500) | G (FLK, pcadapt, FST) | Missense_variant [932] Ser > Asn | Histidine kinase 3 (AHK3) | 72 | 0 | Q9C5U1 | AHK3 acts in the transmission of drought, salt, and cold stress signals ^23,24^. It is involved in many developmental processes: meristem establishment in seedlings, seed germination, cell division, seed size, chlorophyll retention during leaf senescence, root repression and shoot promotion, and flower development ^25–27^. | Stress (salt, drought, cold), Development (flower, plant) |
| Pst_8971 (C > T) | NC_035176.1 (24448370) | LOC110604012 (Manes.16G087200) | G (FLK, pcadapt) | Intron variant | 3beta-hydroxysteroid-dehydrogenase/decarboxylase isoform 2 (3BETAHSD/D2) | 71.9 | 0 | Q67ZE1 | 3BETAHSD/D2 is involved in the regulation of inflorescence internodes and leaf growth ^28^. | Growth (inflorescence, leaf) |
| Pst_29 (T > C) | NC_035161.1 (1823645) | LOC110613144 (Manes.01G010300) | B (hapFLK, FST) | Synonymous variant [132] Val > Val | Thiocyanate methyltransferase 1 (TMT1) | 60.2 | 8.2x10^-70^ | Q93V78 | TMT1 is a methyltransferase possibly involved in glycosinolate metabolism after tissue damage ^29^. | Cyanide, Defense |
| Nsi_2289 (A > G) | NC_035168.1 (30311331) | LOC110620226 (Manes.08G136900) | G (hapFLK, pcadapt) | Intron variant | GRF1-interacting factor 1 (GIF1) | 62.5 | 1.7x10^-62^ | Q8L8A5 | GIF1 is a transcription coactivator involved in the regulation of cell expansion in leaves and cotyledons ^30^, acting in the development of appropriate leaf size and shape. It also mediates cotyledon identity ^31^. | Development (leaf, cotyledon) |
| Nsi_3540 (C > A) | NC_035174.1 (731020) | LOC110599603 (Manes.14G007100) | G (FLK, hapFLK, pcadapt, FST) | Missense variant [147] Ser > Arg | FT-interacting protein 3 (FTIP3) | 60.2 | 0 | Q9M2R0 | FTIP3 is required for the proliferation and differentiation of shoot stem cells in the shoot apical meristem ^32^. | Development of shoot |
| Pst_9853 (A > G) | NC_035178.1 (7429534) | LOC110606992 (Manes.18G086300) | G (FLK, pcadapt) | Intron variant | Transcriptional corepressor SEUSS (SEU) | 62.9 | 0 | Q8W234 | SEU is involved in the transcriptional repression of the C class floral homeotic gene AGAMOUS during flower development ^33^, and in the regulation of petal shape and ovule development ^34^. | Flowering, Transcription regulation |
| Pst_9959 (A > G) | NC_035178.1 (11748439) | LOC110607096 (Manes.18G120200) | B (FLK, hapFLK) | Missense variant [81] Asp > Gly | Agamous-like MADS-box protein AGL12 (AGL12) | 60.4 | 2.8x10^-78^ | Q38841 | AGL12 may be a transcription activator involved in the regulation of root development by controlling cell proliferation. May act as promoter of flowering transition ^35^. | Root, Flowering, Transcription regulation |

a: SNP identification as specified in the vcf file; b: chromosome names are coded according to the manioc genome *Manihot esculenta v6* (NCBI PRJNA234389) and the SNP positions are within parenthesis; c: asterisks indicate putative manioc resistance genes (PRGdb v.3.0); d: B indicates that the tests were performed considering the groups of varieties per biome, while G the groups of wild and cultivated manioc; e: mutational effect predicted for the primary transcript of the associated manioc genes. Amino acid substitutions are coded as [number of the residue] original amino acid > new amino acid; f: E-value = probability of hits by chance; g: Recovered from the UniProt data base (www.unipr ot.org).

**References used in Appendix 1**

1. Mackey, D., Belkhadir, Y., Alonso, J. M., Ecker, J. R. & Dangl, J. L. *Arabidopsis* RIN4 is a target of the type III virulence effector AvrRpt2 and modulates RPS2-mediated resistance. *Cell* **112**, 379–389 (2003).

2. Mackey, D., Holt, B. F., Wiig, A. & Dangl, J. L. RIN4 interacts with *Pseudomonas syringae* type III effector molecules and is required for RPM1-mediated resistance in *Arabidopsis*. *Cell* **108**, 743–754 (2002).

3. Escobar-Restrepo, J.-M. *et al.* The FERONIA receptor-like kinase mediates male-female interactions during pollen tube reception. *Science (80-. ).* **317**, 656–660 (2007).

4. Guo, H. *et al.* Three related receptor-like kinases are required for optimal cell elongation in *Arabidopsis thaliana*. *Proc. Natl. Acad. Sci. USA* **106**, 7648–7653 (2009).

5. Kessler, S. A. *et al.* Conserved molecular components for pollen tube reception and fungal invasion. *Science (80-. ).* **330**, 968–971 (2010).

6. Bleckmann, A., Weidtkamp-Peters, S., Seidel, C. A. M. & Simon, R. Stem cell signaling in Arabidopsis requires CRN to localize CLV2 to the plasma membrane. *Plant Physiol.* **152**, 166–176 (2010).

7. Lukowitz, W., Roeder, A., Parmenter, D. & Somerville, C. A MAPKK kinase gene regulates extra-embryonic cell fate in *Arabidopsis*. *Cell* **116**, 109–119 (2004).

8. Hwang, I. *et al.* Over-expression of the *IGI1* leading to altered shoot-branching development related to MAX pathway in *Arabidopsis*. *Plant Mol. Biol.* **73**, 629–641 (2010).

9. Lamberti, G., Gügel, I. L., Meurer, J., Soll, J. & Schwenkert, S. The cytosolic kinases STY8, STY17, and STY46 are involved in chloroplast differentiation in Arabidopsis. *Plant Physiol.* **157**, 70–85 (2011).

10. Martin, T. *et al.* A protein kinase family in *Arabidopsis* phosphorylates chloroplast precursor proteins. *J. Biol. Chem.* **281**, 40216–40223 (2006).

11. Ueda, M. *et al.* The *HALTED ROOT* gene encoding the 26S proteasome subunit RPT2a is essential for the maintenance of *Arabidopsis* meristems. *Development* **131**, 2101–2111 (2004).

12. Sonoda, Y. *et al.* Regulation of leaf organ size by the Arabidopsis RPT2a 19S proteasome subunit. *Plant J.* **60**, 68–78 (2009).

13. Ueda, M. *et al.* Arabidopsis *RPT2a* encoding the 26S proteasome subunit is required for various aspects of root meristem maintenance, and regulates gametogenesis redundantly with its homolog, *RPT2b*. *Plant Cell Physiol.* **52**, 1628–1640 (2011).

14. Sako, K. *et al.* Arabidopsis RPT2a, 19S proteasome subunit, regulates gene silencing via DNA methylation. *PLoS One* **7**, e37086 (2012).

15. Sakamoto, T. *et al.* *Arabidopsis thaliana* 26S proteasome subunits RPT2a and RPT5a are crucial for zinc deficiency-tolerance. *Biosci. Biotechnol. Biochem.* **75**, 561–567 (2011).

16. Yao, C., Wu, Y., Nie, H. & Tang, D. RPN1a, a 26S proteasome subunit, is required for innate immunity in Arabidopsis. *Plant J.* **71**, 1015–1028 (2012).

17. An, Z. *et al.* Regulation of the stability of RGF1 receptor by the ubiquitin-specific proteases UBP12/UBP13 is critical for root meristem maintenance. *Proc. Natl. Acad. Sci. USA* **115**, 1123–1128 (2018).

18. Okushima, Y., Fukaki, H., Onoda, M., Theologis, A. & Tasaka, M. ARF7 and ARF19 regulate lateral root formation via direct activation of *LBD/ASL* genes in *Arabidopsis*. *Plant Cell* **19**, 118–130 (2007).

19. Wang, X., Jing, Y., Zhang, B., Zhou, Y. & Lin, R. Glycosyltransferase-like protein ABI8/ELD1/KOB1 promotes *Arabidopsis* hypocotyl elongation through regulating cellulose biosynthesis. *Plant, Cell Environ.* **38**, 411–422 (2015).

20. Brocard-Gifford, I., Lynch, T. J., Garcia, M. E., Malhotra, B. & Finkelstein, R. R. The *Arabidopsis thaliana* *Abscisic Acid-Insensitive8* locus encodes a novel protein mediating abscisic acid and sugar responses essential for growth. *Plant Cell* **16**, 406–421 (2004).

21. Lertpiriyapong, K. & Sung, Z. R. The *elongation defective1* mutant of *Arabidopsis* is impaired in the gene encoding a serine-rich secreted protein. *Plant Mol. Biol.* **53**, 581–595 (2003).

22. Wattebled, F. *et al.* Further evidence for the mandatory nature of polysaccharide debranching for the aggregation of semicrystalline starch and for overlapping functions of debranching enzymes in Arabidopsis leaves. *Plant Physiol.* **148**, 1309–1323 (2008).

23. Tran, L. S. P. *et al.* Functional analysis of AHK1/ATHK1 and cytokinin receptor histidine kinases in response to abscisic acid, drought, and salt stress in *Arabidopsis*. *Proc. Natl. Acad. Sci. USA* **104**, 20623–20628 (2007).

24. Jeon, J. *et al.* A subset of cytokinin two-component signaling system plays a role in cold temperature stress response in *Arabidopsis*. *J. Biol. Chem.* **285**, 23371–23386 (2010).

25. Higuchi, M. *et al.* *In planta* functions of the *Arabidopsis* cytokinin receptor family. *Proc. Natl. Acad. Sci. USA* **101**, 8821–8826 (2004).

26. Li, X. G. *et al.* Cytokinin overproduction-caused alteration of flower development is partially mediated by *CUC2* and *CUC3* in Arabidopsis. *Gene* **450**, 109–120 (2010).

27. Riefler, M., Novak, O., Strnad, M. & Schmülling, T. *Arabidopsis* cytokinin receptors mutants reveal functions in shoot growth, leaf senescence, seed size, germination, root development, and cytokinin metabolism. *Plant Cell* **18**, 40–54 (2006).

28. Kim, B., Kim, G., Fujioka, S., Takatsuto, S. & Choe, S. Overexpression of 3β-hydroxysteroid dehydrogenases/C-4 decarboxylases causes growth defects possibly due to abnormal auxin transport in Arabidopsis. *Mol. Cells* **34**, 77–84 (2012).

29. Attieh, J., Sparace, S. A. & Saini, H. S. Purification and properties of multiple isoforms of a novel thiol methyltransferase involved in the production of volatile sulfur compounds from *Brassica oleracea*. *Arch. Biochem. Biophys.* **380**, 257–266 (2000).

30. Fujikura, U., Horiguchi, G., Ponce, M. R., Micol, J. L. & Tsukaya, H. Coordination of cell proliferation and cell expansion mediated by ribosome-related processes in the leaves of *Arabidopsis thaliana*. *Plant J.* **59**, 499–508 (2009).

31. Kanei, M., Horiguchi, G. & Tsukaya, H. Stable establishment of cotyledon identity during embryogenesis in *Arabidopsis* by *ANGUSTIFOLIA3* and *HANABA TARANU*. *Development* **139**, 2436–2446 (2012).

32. Liu, L. *et al.* FTIP-dependent STM trafficking regulates shoot meristem development in *Arabidopsis*. *Cell Rep.* **23**, 1879–1890 (2018).

33. Sridhar, V. V, Surendrarao, A., Gonzalez, D., Conlan, R. S. & Liu, Z. Transcriptional repression of target genes by LEUNIG and SEUSS, two interacting regulatory proteins for *Arabidopsis* flower development. *Proc. Natl. Acad. Sci. USA* **101**, 11494–11499 (2004).

34. Bao, F., Azhakanandam, S. & Franks, R. G. *SEUSS* and *SEUSS-LIKE* transcriptional adaptors regulate floral and embryonic development in Arabidopsis. *Plant Physiol.* **152**, 821–836 (2010).

35. Tapia-López, R. *et al.* An *AGAMOUS*-related MADS-box gene, *XAL1* (*AGL12*), regulates root meristem cell proliferation and flowering transition in Arabidopsis. *Plant Physiol.* **146**, 1182–1192 (2008).

**Supplementary Tables**

**Table S1** – Passport data for the 78 manioc (*Manihot esculenta*) varieties conserved at the Luiz de Queiróz College of Agriculture gene bank, Piracicaba, São Paulo, Brazil, and information about 14 additional samples of cultivated and wild manioc sampled in a previous study (Alves-Pereira *et al*., 2020). Ancestry coefficients for each sNMF genetic cluster considering K =2, 3, and 5, and DAPC membership scores considering the analyses for the biomes plus wild manioc, and only for the biomes are included in the last columns. (In a separate Excel file).

**Table S2** – Variant calling format (VCF) file containing information for the 11,782 SNP markers identified for the 92 manioc (*Manihot esculenta*) varieties, based on two double-digest genotyping-by-sequencing genomic libraries. (In a separate Excel file).

**Table S3** – Predicted effects of mutations associated with the 865 SNP markers putatively under selection. CHROM = chromosome identification, and POS = position of the SNP in the manioc genome coded according to *Manihot esculenta v6* (NCBI PRJNA234389). ID = identification of the SNP marker; REF = reference allele; ALT = alternative allele; ANN = annotation of the predicted effects. Predicted effects on more than one gene transcript or in downstream/upstream genes are specified in different ANN columns. (In a separate Excel file).

**Table S4** – Results from the enrichment analysis based on GO annotations of the 663 manioc predicted genes with SNPs putatively under selection. GO annotations and their descriptions are shown with their respective observed and expected counts, and associated p-values from Fisher’s exact test (In a separate Excel file).

**Table S5 –** Summary of blastp results for 337 amino acid sequences from manioc genes (*Manihot esculenta v6*, NCBI PRJNA234389) with outlier SNPs. Similarities are shown for 45 amino acid sequences with identity > 90 % when compared to the predicted resistance genes for manioc (PRGdb v.3.0) and 306 amino acid sequences with identity > 60 % with proteins deposited in Swiss-Prot. A summary of putative gene functions is also present if available in UniProt (www.unipr ot.org). (In a separate Excel file).

**Table S6** – Comparison of genetic diversity and inbreeding estimates based on 10,917 neutral SNPs for the groups of 92 manioc varieties and the 78 varieties conserved in the gene bank (within brackets). The groups of cultivated maniocs, and each distinct biome include varieties for which there are no information about reputed toxicity (non-designated) in the gene bank passport data. Number of samples (N), Total number of alleles (A), percentage of polymorphic loci (%P), number of private alleles (PA), observed (*H_O_*) and expected (*H_E_*) heterozygosities, inbreeding coefficients (*f*), and 95 % confidence intervals (95%CI).

| Groups | N | *A* | *%P* | *PA* | *H_O_* (95%CI) | *H_E_* (95%CI) | *f* (95%CI) |
| --- | --- | --- | --- | --- | --- | --- | --- |
| Cultivated | 84 | 21,516 | 98.5 | 7054 | 0.324 (0.321; 0.328) | 0.315 (0.312; 0.318) | -0.030 (-0.071; 0.005) |
|  | [78] | [21,487] | [98.4] | [7054] | [0.328 (0.324; 0.331)] | [0.313 (0.310; 0.316)] | [-0.046 (-0.089; -0.010)] |
| Bitter | 16 | 21,009 | 97.9 | 60 | 0.295 (0.291; 0.298) | 0.309 (0.306; 0.312) | 0.047 (-0.014; 0.093) |
|  | [13] | [20,937] | [97.7] | [74] | [0.302 (0.297; 0.304)] | [0.309 (0.306; 0.312)] | [0.026 (-0.052; 0.081)] |
| Sweet | 43 | 21,435 | 98.2 | 43 | 0.322 (0.318; 0.326) | 0.308 (0.305; 0.310) | -0.047 (-0.105; -0.001) |
|  | [40] | [21,370] | [97.9] | [38] | [0.325 (0.321; 0.328)] | [0.306 (0.303; 0.309)] | [-0.061 (-0.126; -0.011)] |
| Wild | 8 | 14,780 | 67.7 | 318 | 0.103 (0.098; 0.105) | 0.122 (0.118; 0.125) | 0.177 (-0.040; 0.354) |
| Biomes |  |  |  |  |  |  |  |
| Amazonia | 22 | 21,240 | 98.8 | 153 | 0.293 (0.290; 0.296) | 0.312 (0.310; 0.315) | 0.062 (0.015; 0.097) |
|  | [16] | [21,137] | [98.4] | [124] | [0.299 (0.295; 0.302)] | [0.312 (0.310; 0.315)] | [0.044 (-0.021; 0.093)] |
| Cerrado | 30 | 21,108 | 98.2 | 16 | 0.342 (0.337; 0.346) | 0.298 (0.295; 0.301) | -0.145 (-0.216; -0.090) |
| Atlantic Forest | 27 | 21,142 | 99.7 | 7 | 0.325 (0.321; 0.329) | 0.300 (0.297; 0.303) | -0.085 (-0.167; -0.026) |
| Pantanal | 5 | 19,604 | 92.7 | 0 | 0.349 (0.344; 0.354) | 0.285 (0.281; 0.288) | -0.226 (-0.492; -0.107) |

**Table S7** – Comparison of analyses of molecular variance based on 10,917 neutral SNPs, showing the genetic variation within and among hierarchical groups, considering all the 92 varieties and the 78 varieties conserved in the gene bank (within brackets). All the analyses, except among bitter, sweet, and wild, include varieties for which there are no information about reputed toxicity (non-designated) in the gene bank passport data. Degrees of freedom (Df).

| Source of variation | Df | Sum of squares | Variance components | Percentage of variance | φ-statistics |
| --- | --- | --- | --- | --- | --- |
| Between Wild and Cultivated | 1  [1] | 22,823.83  [23,022.38] | 729.25  [740.41] | 32.5  [32.6] | φ_ST_ = 0.32 (p < 0.001)  [0.326 (p < 0.001)] |
| Within Wild and Cultivated | 182  [170] | 276,088.28  [260,663.40] | 1,516.97  [1,533.31] | 67.5  [67.4] |  |
| Total | 183  [171] | 298,912.11  [283,685.78] | 2,246.22  [2,273.72] |  |  |
|  |  |  |  |  |  |
| Among Bitter, Sweet, and Wild | 2  [2] | 26,517.04  [26,174.50] | 341.13  [375.32] | 19.1  [20.3] | φ_ST_ = 0.19 (p < 0.001)  [0.203 (p < 0.001)] |
| Within Bitter, Sweet, and Wild | 131  [119] | 189,459.88  [175,011.93] | 1,446.26  [1,470.68] | 80.9  [79.7] |  |
| Total | 133  [121] | 215,976.92  [201,186.43] | 1,787.39  [1,846.01] |  |  |
|  |  |  |  |  |  |
| Among Biomes | 3  [3] | 11,073.90  [9,589.59] | 54.76  [45.18] | 3.4  [2.8] | φ_ST_ = 0.03 (p < 0.001)  [0.028 (p < 0.001)] |
| Within Biomes | 164  [152] | 254,820.13  [240,884.75] | 1,553.78  [1,584.77] | 96.6  [97.2] |  |
| Total | 167  [155] | 265,894.03  [250,474.34] | 1,608.54  [1,629.95] |  |  |
|  |  |  |  |  |  |
| Between Biomes and Wild | 1  [1] | 22,823.83  [23,022.38] | 693.60  [710.26] | 31.1  [31.4] | φ_ST_ = 0.34 (p < 0.001)  [0.335 (p < 0.001)] |
| Among groups within Biomes and Wild | 3  [3] | 11,073.90  [9,589.59] | 56.64  [47.46] | 2.5  [2.1] | φ_SC_ = 0.03 (p < 0.001)  [0.003 (p < 0.001)] |
| Within Biomes and Wild | 179  [167] | 265,014.38  [251,073.81] | 1,480.53  [1,503.44] | 66.4  [66.5] | φ_CT_ = 0.31 (p = 0.19)  [0.31 (p = 0.20)] |
| Total | 183  [171] | 298,912.11  [283,685.78] | 2,230.77  [2,261.15] |  |  |

**Table S8** – Comparison of pairwise estimates of genetic divergence (Weir & Cockerham’s F_ST_ (1984)) based on 10,917 neutral SNPs, considering all the 92 varieties and the 78 varieties conserved in the gene bank (within brackets). Each biome also includes varieties for which there are no information about reputed toxicity (non-designated) in the gene bank passport data. Asterisks indicate estimates significant at p < 0.01.

| Groups | Bitter | Sweet |  | Biomes | Amazonia | Cerrado | Atlantic Forest | Pantanal |
| --- | --- | --- | --- | --- | --- | --- | --- | --- |
| Sweet | 0.045* [0.044*] |  |  | Cerrado | 0.065* [0.060*] |  |  |  |
| Wild | 0.357* [0.349*] | 0.344* [0.349*] |  | Atlantic Forest | 0.045* [0.039*] | 0.017 |  |  |
|  |  |  |  | Pantanal | 0.044* [0.036] | -0.025 | -0.015 |  |
|  |  |  |  | Wild | 0.342* [0.339*] | 0.365* | 0.362* | 0.433* |

**Supplementary Figures**


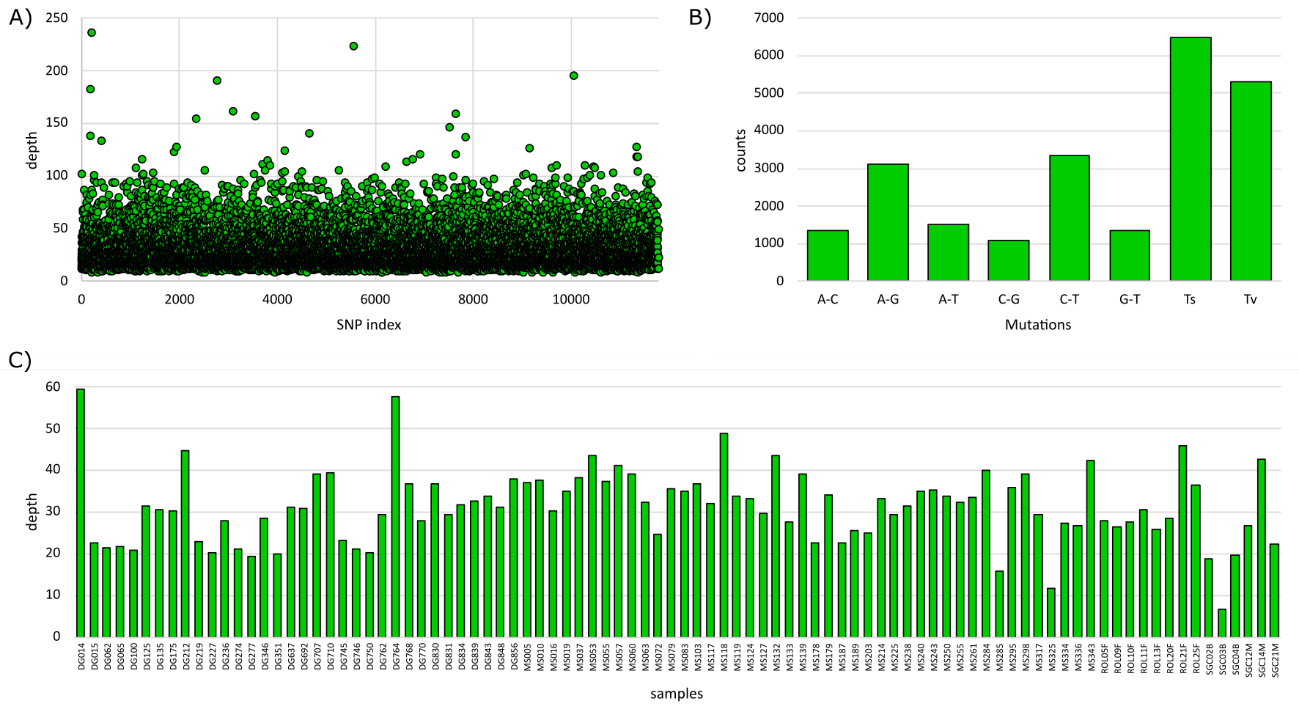


**Figure S1** – Quality metrics of the 11,782 SNP markers identified for 92 manioc (*Manihot esculenta*) varieties based on two double-digest genotyping-by-sequencing genomic libraries. A) mean sequence depth per locus, B) count of mutations (Ts = transitions, Tv = transversions), and C) mean sequence depth per sample.


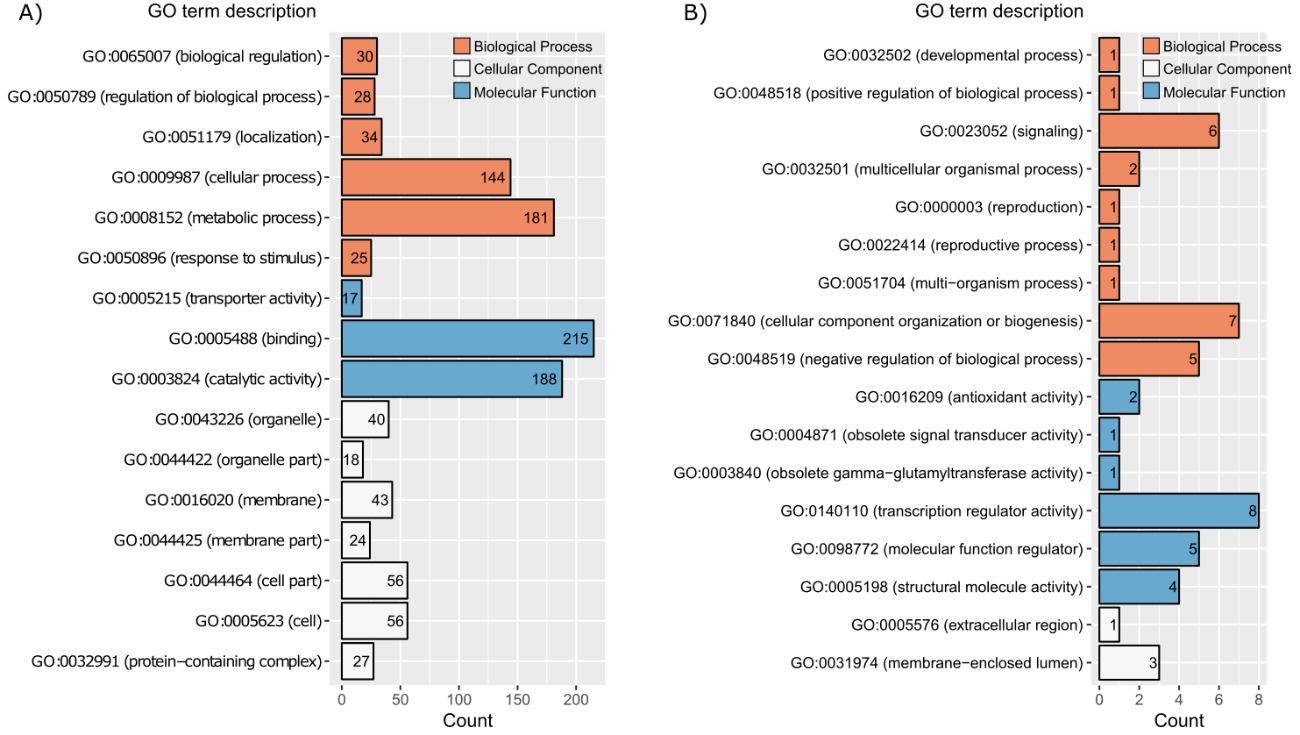


**Figure S2** – Summary of GO annotations of the 663 manioc predicted genes with SNPs putatively under selection. The annotations are grouped according to their biological processes, molecular functions, or cellular components. To facilitate visualization, bar plots were split for the GO terms occurring more than (A) or less than (B) 10 times. The number of genes with each annotation is indicated inside the bars.


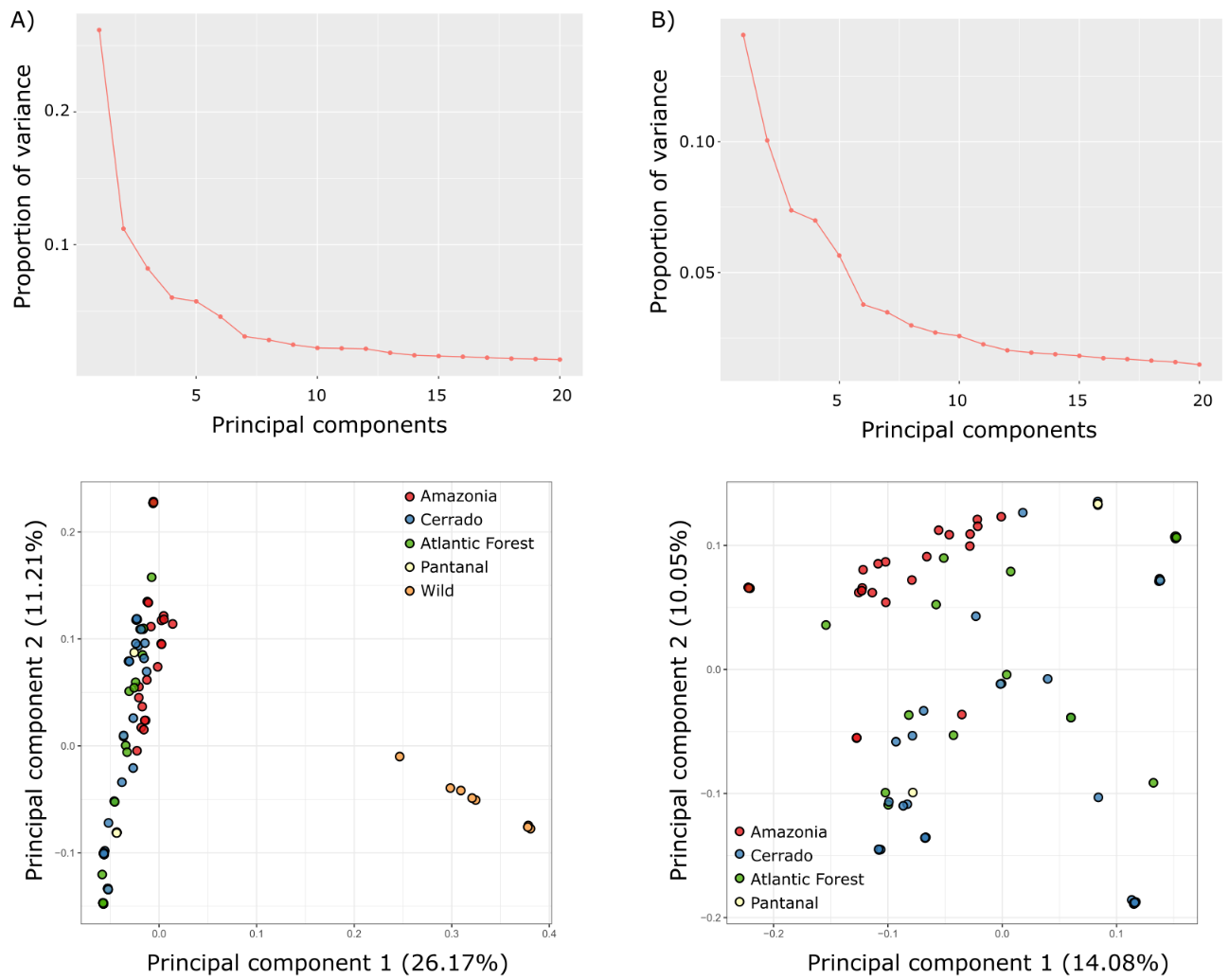


**Figure S3** – Pcadapt results for the detection of outlier SNPs considering A) the groups of wild versus cultivated manioc (*Manihot esculenta*, N = 92), and B) the groups of varieties per biomes (without wild samples, N = 84). The analyses were performed based on 11,782 SNP markers. Top: Scree plots of the proportion of explained variance in the principal component analysis (PCA) for the first K = 20 principal components. The number of components retained in the analyses followed Cattle’s rule, choosing the point to the left where the curve inflects, and the addition of components does not substantially increase the amount of variance explained. We chose K = 2 for the analysis contrasting wild and cultivated manioc, and K = 5 for the analysis contrasting the biomes. Bottom: Scatter plots of the first two principal components showing the major genetic structure observed in the PCAs for the two groupings of samples.
